# Sciatica and Mental Well-Being Among Saudi Women: A Cross-Sectional Investigation

**DOI:** 10.3390/healthcare14091227

**Published:** 2026-05-02

**Authors:** Mohammad A. Jareebi

**Affiliations:** Department of Family and Community Medicine, College of Medicine, Jazan University, Jazan 45142, Saudi Arabia; mjareebi@jazanu.edu.sa

**Keywords:** sciatica, stress, mental health, depression, anxiety, DASS-21, women’s health, Saudi Arabia, chronic pain, psychosocial factors

## Abstract

Background/Objectives: Sciatica can adversely affect mental well-being; however, evidence regarding its psychological impact among Saudi women remains scarce, particularly concerning differential effects across specific mental health domains. This study examined the prevalence of sciatica and its associations with depression, anxiety, and stress among adult Saudi women. Methods: A cross-sectional online survey was conducted from February to March 2024 among Saudi women aged ≥18 years. Participants (n = 706) completed the Arabic Depression, Anxiety, and Stress Scale (DASS-21) and provided sociodemographic and health information. Sciatica status was based on self-report. Multivariable linear regression analyses identified independent predictors of each mental health domain. Results: Sciatica prevalence was 11.0% among 706 participants (mean age 29 ± 11 years; mean BMI 24 ± 6.5 kg/m^2^). Sciatica was the strongest independent predictor of stress (β = 6.87, 95% CI: 4.57–9.17, *p* < 0.001). No significant associations were observed with depression (β = 1.80, *p* = 0.183) or anxiety (β = 0.45, *p* = 0.481). Additional stress predictors included lower-back pain, diabetes, lower–middle income, and daily phone use >8 h, while bachelor-level education was protective. Arthritis independently predicted anxiety (β = 1.52, *p* = 0.008). Conclusions: In this convenience sample of Saudi women, sciatica was significantly associated with higher stress symptom scores, while associations with depression and anxiety did not reach statistical significance. The observed pattern suggests that stress screening and management should be considered within biopsychosocial care for sciatica patients, pending confirmation in prospective studies.

## 1. Introduction

The sciatic nerve, formed from the L4 to S3 spinal nerve roots, is the largest nerve in the human body and a major branch of the sacral plexus [1]. Sciatica, clinically referred to as lumbar radicular pain, arises from dysfunction or irritation of the sciatic nerve or its roots, with both nerve root compression and inflammation necessary for symptom development [2]. Epidemiological studies indicate that 13–40% of individuals may experience sciatica at some point in their lives, with an annual incidence ranging from 1% to 5% [3,4].

Sciatica typically manifests as sharp, radiating pain extending from the lower back down the leg, often accompanied by numbness, tingling, and pain exacerbation during specific movements [5,6]. The most common cause is nerve root compression at the L4/L5 level due to lumbar disk herniation, though other contributing factors include muscle spasms, postural abnormalities, and sustained mechanical stress [7]. Occupational factors significantly influence sciatica risk, with manual laborers and individuals engaged in prolonged trunk flexion, twisting, or prolonged driving exhibiting higher rates [8]. While most acute cases resolve with conservative management, 20–30% of patients experience persistent symptoms beyond one year [9].

In Saudi Arabia, where rapid modernization has introduced new health challenges, low back pain prevalence ranges between 63.8% and 89% [10], yet data on sciatica and its specific psychological impact remain limited [11]. Beyond physical disability, sciatica exerts a substantial psychological burden. Recently, we demonstrated that sciatica significantly impairs overall quality of life among Saudi adults, with chronic sciatica (>1 year duration) showing particularly strong associations with poor wellbeing (OR = 0.29, *p* = 0.006) [12]. However, quality of life represents a broad construct encompassing physical, mental, social, and emotional dimensions. It remains unclear which specific psychological domains such as depression, anxiety, or stress drive the mental health burden of sciatica, or whether the condition exhibits a differential psychological profile rather than generalized mood disturbance. Mental wellbeing encompasses emotional, psychological, and social functioning, and its impairment in chronic pain is well-documented. Studies report elevated depression in 30–45% and anxiety in 20–40% of individuals with chronic radicular pain [13]. Rayner et al. (2016) demonstrated that chronic pain patients show significantly poorer psychological wellbeing compared to pain-free individuals, independent of physical disability [14].

Understanding this distinction has critical clinical implications. Generic mental health screening may miss condition-specific patterns, and interventions targeting one psychological domain (e.g., depression) may prove ineffective if the primary burden manifests through another pathway. Depression may develop through learned helplessness and loss of function; anxiety through fear-avoidance and hypervigilance to pain signals; and stress through immediate adaptive demands of managing unpredictable pain alongside daily roles [15]. Chronic pain conditions often produce varied psychological profiles. The existing literature reports mixed findings across depression, anxiety, and stress domains: some chronic pain populations show predominantly depressive responses, others exhibit heightened anxiety, and others show elevations across all three dimensions [13]. No a priori assumption can be made about which domain is most affected by sciatica specifically.

Gender-specific analysis is particularly warranted given well-documented sex differences in pain processing, psychological comorbidity, and pain-related coping strategies [16]. Women demonstrate higher prevalence of anxiety and depression, greater pain sensitivity, and distinct patterns of catastrophizing and help-seeking behaviors compared to men [17]. Parity was examined as a demographic covariate given that pregnancy-related sciatica resulting from fetal pressure on the sciatic nerve or pelvic muscle spasm, is a recognized, typically transient, phenomenon [7], and multiparity is common in this population [18].

Despite the high prevalence of musculoskeletal conditions in Saudi Arabia, the relationship between sciatica and specific mental health dimensions—depression, anxiety, and stress—remains unexplored in this population, particularly among women. To address this critical gap, this study aimed to (1) determine the prevalence of sciatica among adult Saudi females, (2) assess its differential impact on depression, anxiety, and stress as measured by the Depression, Anxiety and Stress Scale (DASS-21), and (3) identify key factors, including pregnancy history, influencing these psychological outcomes. We hypothesized that sciatica would be associated with poorer mental health outcomes across at least one psychological domain. The differential impact across depression, anxiety, and stress domains was treated as an open empirical question given the absence of prior evidence in this population.

## 2. Materials and Methods

### 2.1. Study Design and Setting

This cross-sectional study was conducted between February and March 2024 among adult Saudi females residing in Saudi Arabia. Data collected electronically using a structured, self-administered Arabic questionnaire hosted on Google Forms (Google Forms (Google LLC, Mountain View, CA, USA; latest cloud version) was used to develop the survey. The survey link was disseminated through major social media platforms, including WhatsApp (version 2.26.16.72; WhatsApp LLC, Menlo Park, CA, USA), X/Twitter (version 7.82.0-release.32; X Corp., San Francisco, CA, USA), Telegram (version 12.6.4; Telegram FZ-LLC, Dubai, United Arab Emirates), and Facebook (latest app version; Meta Platforms, Inc., Menlo Park, CA, USA), to ensure wide accessibility and maximize participation across different regions [19]. Respondents completed the questionnaire at their convenience, ensuring anonymity and voluntary participation. Only participants who satisfied all eligibility criteria were included in the analysis. The minimum required sample size was calculated as 461 participants, based on the regional adult female population (approximately 460,000), a 95% confidence interval (*Z* = 1.96), a 5% margin of error, an estimated prevalence of 50% (conservative estimate for maximum variability), and accounting for a 20% nonresponse rate. The following formula was applied [20].

$$n=\frac{\left(Z^{2}\;\times \;P\;\times \;\left(1-P\right)\right)}{d^{2}}$$

where *Z* = 1.96 (95% confidence level), *P* = 0.50, and *d* = 0.05 (5% margin of error). A total of 706 participants were ultimately recruited, exceeding the minimum requirement and enhancing statistical power.

### 2.2. Eligibility Criteria

Inclusion criteria required participants to be Saudi female nationals aged 18 years or older, capable of reading and understanding Arabic, and willing to provide informed consent. Exclusion criteria eliminated non-Saudi nationals and individuals with severe cognitive impairments that might hinder accurate questionnaire completion.

### 2.3. Data Collection Tool

The questionnaire comprised three main sections. First, sociodemographic information was collected, including age, height, weight, residence (urban/village), education level (secondary school or less, bachelor’s degree, postgraduate), marital status (single, married, widowed/divorced), employment status, and monthly family income. Second, health-related and lifestyle characteristics were assessed, including smoking status, physical activity engagement, presence of chronic conditions (diabetes, arthritis), daily phone usage duration, daily driving duration, and pregnancy history (for ever-married participants). Sciatica-related variables included self-reported physician diagnosis, symptom duration (<1 year, >1 year), history of lower back pain (LBP), and family history of sciatica. Third, mental health outcomes were assessed using the Arabic version of the Depression, Anxiety, and Stress Scale (DASS-21).

DASS-21 is a validated 21-item instrument comprising three subscales (depression, anxiety, stress) with seven items each [21]. Participants rated each item on a 4-point Likert scale ranging from 0 (“did not apply to me at all”) to 3 (“applied to me very much, or most of the time”). Subscale scores were calculated by summing the seven items and multiplying by 2, yielding scores ranging from 0 to 42 for each dimension. Established cutoff scores were used to categorize severity: depression (normal: 0–9, mild: 10–13, moderate: 14–20, severe: 21–27, extremely severe: ≥28), anxiety (normal: 0–7, mild: 8–9, moderate: 10–14, severe: 15–19, extremely severe: ≥20), and stress (normal: 0–14, mild: 15–18, moderate: 19–25, severe: 26–33, extremely severe: ≥34). DASS-21 has demonstrated excellent psychometric properties in Arabic-speaking populations, with Cronbach’s alpha coefficients ranging from 0.88 to 0.94 across subscales [22,23].

### 2.4. Statistical Analysis

Data were analyzed using R software (version 4.2.3, R Foundation for Statistical Computing, Vienna, Austria). Continuous variables were expressed as mean ± standard deviation (SD), and categorical variables as frequencies and percentages.

Univariate analyses were conducted to examine bivariate associations between key independent variables (sciatica diagnosis, lower back pain, diabetes, arthritis, education level, family income, daily phone use) and each mental health outcome (depression, anxiety, stress scores). Independent samples *t*-tests were used for binary variables, and one-way analysis of variance (ANOVA) for categorical variables with more than two levels.

Multivariable linear regression analyses were then performed to identify independent predictors of each DASS-21 subscale score. All potential predictors were entered simultaneously into separate models for depression, anxiety, and stress. These included sciatica diagnosis, lower back pain, age, body mass index (BMI), residence, education level, marital status, employment status, family income, smoking status, physical activity, daily phone use, daily driving duration, pregnancy history, diabetes, and arthritis. Statistical significance was determined at α = 0.05, and 95% confidence intervals (CIs) were reported for all effect estimates. For each multivariable linear regression model, standard diagnostic checks were performed, including examination of residual plots for normality and homoscedasticity, assessment of influential observations via Cook’s distance, and variance inflation factors (VIF) to detect multicollinearity; no substantial violations were identified. Missing data was minimal across all variables and complete case analysis was performed. Given that three separate outcome models were estimated with multiple predictors, the risk of false-positive findings is acknowledged; all results should therefore be interpreted as exploratory and hypothesis-generating rather than confirmatory, and replication in independent samples is recommended. All analyses are adhered to the Strengthening Reporting of Observational Studies in Epidemiology (STROBE) guidelines [24]. No formal statistical comparison was made between regression coefficients across the three outcome models; observed differences in the pattern of significant and non-significant findings should therefore be interpreted descriptively rather than as evidence of a formally established differential association.

### 2.5. Ethical Approval

The study protocol received ethical approval from the Research Ethics Committee at Jazan University (Reference number: REC-45/07/973, dated 7 February 2024). The study was conducted in accordance with the Declaration of Helsinki and institutional ethical standards. Electronically informed consent was obtained from all participants after they were informed about the study’s purpose, procedures, voluntary participation, and confidentiality measures. Data was anonymized, and no personal identifiers were collected or stored.

### 2.6. Use of Generative Artificial Intelligence

Generative AI (GPT-5, OpenAI) was used solely for language editing. Study design, data, analysis, and interpretation were entirely conducted by the author.

## 3. Results

### 3.1. Participant Characteristics

A total of 706 female participants were enrolled, with a mean age of 29 ± 11 years and a mean BMI of 24 ± 6.5 kg/m^2^. The majority resided in villages (56.8%, n = 401) and held a bachelor’s degree (76.8%, n = 542). Most participants were single (57.1%, n = 403), followed by married (38.5%, n = 272). Nearly half were students (48.3%, n = 435), while 23.4% (n = 165) worked in the governmental sector. Family income distribution was as follows: 35.4% (n = 250) earned less than 5000 SAR, 19.8% (n = 140) earned 5000–9999 SAR, 20.4% (n = 144) earned 10,000–14,999 SAR, and 24.4% (n = 172) earned more than 15,000 SAR (Table 1).

### 3.2. Health-Related and Lifestyle Characteristics

Most participants were non-smokers (97.6%, n = 689), with only 2.4% (n = 17) reporting current smoking. Physical activity was reported by 58.8% (n = 415) of participants. Pre-existing health conditions were relatively uncommon: 5.5% (n = 39) had diabetes and 17.0% (n = 120) reported arthritis. Daily phone use was high, with 40.9% (n = 289) using phones for 4–8 h and 28.6% (n = 202) for more than 8 h per day. The majority of participants reported being single or having no pregnancies (62.2%, n = 439), while 21.4% (n = 151) had a history of more than three pregnancies (Table 2).

### 3.3. Prevalence of Sciatica and Psychological Outcomes

Based on self-reported physician diagnosis, an estimated 11.0% (n = 78) of participants reported a sciatica diagnosis; however, given the absence of clinical confirmation and the convenience sampling strategy, this figure should be regarded as an indicative estimate rather than a precise epidemiological prevalence. Among those with sciatica, 60.3% (n = 47) reported symptom duration of less than one year, while 39.7% (n = 31) had symptoms persisting for more than one year. Lower back pain (LBP) was reported by 26.3% (n = 186) of participants, and 30.3% (n = 214) had a family history of sciatica (Table 3).

Regarding psychological symptoms, 76.6% (n = 541) of participants reported normal stress levels, while 23.4% (n = 165) experienced mild to extremely severe stress. For anxiety, 58.1% (n = 410) reported normal levels, with 41.9% (n = 296) experiencing some degree of anxiety symptoms. Depression levels were normal in 65.6% (n = 463) of participants, while 34.4% (n = 243) reported mild to extremely severe depression. Notably, extremely severe anxiety was present in 12.3% (n = 87) of participants, and moderate to extremely severe depression affected 25.7% (n = 181) (Table 4).

Severity categories based on DASS-21 cutoff scores: Stress (normal: 0–14, mild: 15–18, moderate: 19–25, severe: 26–33, extremely severe: ≥34); Anxiety (normal: 0–7, mild: 8–9, moderate: 10–14, severe: 15–19, extremely severe: ≥20); Depression (normal: 0–9, mild: 10–13, moderate: 14–20, severe: 21–27, extremely severe: ≥28).

### 3.4. Univariate Associations Between Sciatica and Mental Health Outcomes

Participants with sciatica reported higher stress symptom scores compared to those without sciatica (mean ± SD: 21.4 ± 10.2 vs. 13.8 ± 8.5, *p* < 0.001); these unadjusted findings should be interpreted cautiously as they do not account for potential confounders and are presented for descriptive purposes only. However, no significant differences were observed in depression scores (12.8 ± 9.7 vs. 11.2 ± 8.9, *p* = 0.183) or anxiety scores (10.5 ± 9.3 vs. 9.8 ± 8.7, *p* = 0.481) between the two groups (Table 5).

Additional variables showing significant univariate associations with stress included lower back pain (16.2 ± 9.8 vs. 13.2 ± 8.4, *p* = 0.001), diabetes (18.7 ± 11.2 vs. 13.9 ± 8.8, *p* = 0.004), and arthritis (16.3 ± 10.1 vs. 13.6 ± 8.6, *p* = 0.003). For anxiety, arthritis was associated with higher scores (11.8 ± 9.9 vs. 9.4 ± 8.5, *p* = 0.008). No variables showed significant univariate associations with depression at the *p* < 0.05 level (Table 5).

### 3.5. Multivariable Predictors of Mental Health Outcomes

**Figure 1:**
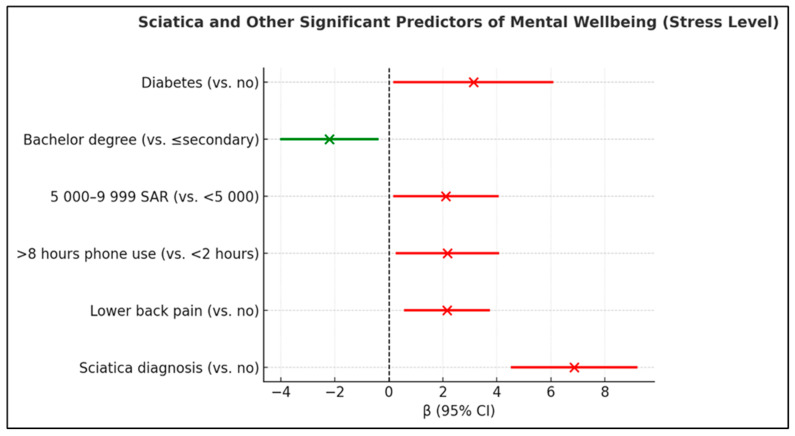
Forest plot illustrating the significant predictors of mental wellbeing (stress level) among participants. Sciatica diagnosis, lower back pain, high daily phone usage, and moderate income were associated with increased stress (red), whereas higher education level was associated with decreased stress (green). Error bars represent 95% confidence intervals for β coefficients.

Multivariable linear regression analysis revealed no significant associations between any examined variables—including sciatica diagnosis, sociodemographic factors, lifestyle behaviors, or comorbidities—and depression scores (all *p* > 0.05) (Complete model results including all predictors are presented in Table A1). Among all variables examined, arthritis was the only significant predictor of anxiety (β = 1.52, 95% CI: 0.39–2.64, *p* = 0.008). Sciatica diagnosis showed no association with anxiety (β = 0.45, 95% CI: −0.80 to 1.70, *p* = 0.481). All other variables, including sociodemographic characteristics, lifestyle factors, and comorbidities, were non-significant (all *p* > 0.05) (Complete model results including all predictors are presented in Table A2). Multivariable linear regression identified sciatica diagnosis as the strongest independent predictor of elevated stress (β = 6.87, 95% CI: 4.57–9.17, *p* < 0.001). Additional significant stress predictors included lower back pain (β = 2.15, 95% CI: 0.60–3.70, *p* = 0.007), diabetes (β = 3.13, 95% CI: 0.21–6.05, *p* = 0.036), lower–middle family income of 5000–9999 SAR (β = 2.11, 95% CI: 0.20–4.03, *p* = 0.031), and daily phone use exceeding 8 h (β = 2.17, 95% CI: 0.29–4.04, *p* = 0.023). In contrast, bachelor’s degree holders were negatively associated with stress (β = −2.21, 95% CI: −3.99 to −0.43, *p* = 0.015), indicating a protective effect. Other examined variables (age, BMI, smoking status, physical activity, residence, marital status, employment) showed no significant associations with stress (Table 6, Figure 1).

## 4. Discussion

### 4.1. Key Findings

This cross-sectional study of 706 Saudi women revealed that sciatica symptom scores were more strongly associated with stress than with depression or anxiety on the DASS-21. Based on self-reported physician diagnosis, 11.0% of participants reported sciatica; however, given the convenience sampling strategy and absence of clinical confirmation, this figure should be regarded as an indicative estimate rather than a precise epidemiological prevalence and interpreted accordingly. Among participants, 11.0% reported sciatica, and multivariable analysis identified it as the strongest independent predictor of elevated stress (β = 6.87, 95% CI: 4.57–9.17, *p* < 0.001). While associations with depression and anxiety were non-significant, these findings do not confirm the absence of an association; the study may have been underpowered to detect smaller effects in these domains. Furthermore, no formal statistical test was conducted to compare the magnitude of associations across the three outcome models; the observed pattern therefore reflects descriptive findings rather than a formally established differential association [25]. Participants with sciatica reported mean stress symptom scores in the mild-to-moderate range (21.4 ± 10.2), whereas those without sciatica scored within the normal range (13.8 ± 8.5); the multivariable-adjusted findings should be considered more reliable than this unadjusted difference. These findings extend our recent demonstration that sciatica significantly impairs overall quality of life in Saudi adults [12], revealing a pattern of higher stress symptom scores among women with sciatica; however, this represents an observed associative pattern from a cross-sectional self-report study and should not be interpreted as evidence of a distinct stress-specific psychological profile.

### 4.2. Possible Explanations for the Pattern of DASS-21 Subscale Differences

DASS-21 subscales are symptom severity scores derived from a self-report instrument and do not represent diagnostically distinct psychological constructs or mechanisms. With this caveat, the pattern of higher stress subscale scores relative to depression and anxiety subscale scores in participants with sciatica is noteworthy; however, non-significant findings for depression and anxiety do not confirm the absence of these associations, and no formal between-outcome comparison was performed to establish that the stress association is statistically superior to the others [13].

Several features of sciatica have been proposed in broader literature that may offer tentative and speculative explanations for this pattern. Sciatica’s episodic and unpredictable nature may generate uncertainty and vigilance, while acute functional limitations may create immediate adaptive demands; however, these explanations are speculative and cannot be confirmed from the present data [26,27]. Such acute functional limitations may specifically generate stressful reactions including heightened emotional distress, fear avoidance behaviors, role disruptions, sleep disruption, adaptive demand for self-care, and social isolation [28]

Although prior literature has proposed neurobiological pathways linking pain chronicity to stress, depression, and anxiety [29,30], no such mechanistic measures were collected in this study; these observations therefore remain speculative and causal or mechanistic conclusions cannot be drawn from the available data. Psychoneurological research suggests that chronic pain-related depression and anxiety involve dysregulation of the hypothalamic–pituitary–adrenal axis, altered prefrontal-limbic connectivity, and reduced hippocampal neurogenesis. While acute stress responses are mediated primarily through sympathetic nervous system activation, pathways operating on different timescales that may explain differential psychological responses at varying stages of pain chronicity [31].

### 4.3. Comorbidities and Psychological Outcomes

Diabetes emerged as a significant stress predictor (β = 3.13, *p* = 0.036), consistent with evidence that women with diabetes experience elevated psychological distress [32,33]. The bidirectional relationship is well-established: stress responses elevate blood glucose through cortisol secretion, while chronic diabetes self-management demands act as persistent psychological stressors [34]. Beyond cortisol-mediated glucose dysregulation, diabetes-related stress encompasses disease management burden, fear of complications, and reduced quality of life, all of which compound sciatica-related psychological distress [35]. Women with both diabetes and chronic pain have been shown to report significantly higher psychological distress scores compared to those with either condition alone [13]. Among our participants, diabetes likely compounds sciatica-related stress through overlapping neuropathic and inflammatory mechanisms alongside the dual self-management burden.

Arthritis was the sole significant predictor of anxiety (β = 1.52, *p* = 0.008) but showed no association with stress, aligning with systematic reviews reporting 20–30% anxiety disorder prevalence in arthritis patients [36,37]. The anxiety associated with arthritis likely reflects heightened interoceptive awareness, fear of disease progression, and uncertainty about functional decline—mechanisms consistent with the fear-avoidance model of chronic musculoskeletal pain [15]. The co-occurrence of arthritis and sciatica in some participants may therefore compound anxiety burden through overlapping fear-avoidance pathways [38]. This differential pattern, arthritis associating with higher anxiety subscale scores and sciatica associating with higher stress subscale scores on the DASS-21, suggests that symptom burden may vary across musculoskeletal conditions, though clinical diagnostic confirmation would be required before inferring distinct psychological disorder profiles.

### 4.4. Socioeconomic and Lifestyle Factors

Lower–middle family income predicted higher stress (β = 2.11, *p* = 0.031), reflecting compounded burdens of financial constraints, healthcare costs, and work capacity concerns [38,39], while bachelor-level education conferred protection (β = −2.21, *p* = 0.015) through enhanced health literacy, coping skills, and social resources [40,41]. Excessive daily phone use (>8 h) also significantly predicted stress (β = 2.17, *p* = 0.023), consistent with evidence linking prolonged screen time to psychological distress through disrupted sleep, reduced social interaction, and cognitive overload [42,43], potentially creating a vicious cycle whereby pain-related mobility restrictions increase phone dependency, further exacerbating stress and postural pain [44]. Together, these findings underscore the role of social determinants in mental health and suggest that interventions addressing financial strain, educational disparities, and digital wellness may reduce psychological burden alongside clinical treatment.

### 4.5. Gender and Cultural Context

Our focus on women addresses a critical gap, as women demonstrate higher pain sensitivity, greater prevalence of anxiety and depression, and distinct psychosocial stressors in the Saudi cultural context [16,17], where traditional caregiving responsibilities, varying social mobility, and potential healthcare barriers may amplify chronic pain’s psychological impact [16]. In contrast, studies in male populations report lower rates of anxiety and depression in response to chronic musculoskeletal pain, with men more likely to employ problem-focused and avoidant coping strategies rather than emotion-focused responses, potentially attenuating psychological symptom expression [17,45]. The absence of a male comparison group in the present study limits direct sex-based comparisons, and this remains an important direction for future research.

Whether cultural and gender-specific contextual factors explain the observed pattern of subscale scores cannot be determined from the present data; these explanations remain speculative and require dedicated measurement in future studies. Although the proportion of multiparous women in our sample (21% with >3 pregnancies) reflects Saudi demographic patterns, pregnancy history showed no significant associations with any mental health outcome in multivariable models, possibly reflecting heterogeneity in pregnancy experiences, varying time since last pregnancy, or insufficient statistical power; clinicians should nonetheless remain attentive to pregnancy-related sciatica as a potential stressor, and future longitudinal studies should examine this relationship more rigorously.

### 4.6. Clinical and Public Health Implications

These findings support several clinical practice changes. First, routine psychological screening for sciatica patients should employ comprehensive tools such as the DASS-21 that capture depression, anxiety, and stress symptoms simultaneously, rather than single-domain instruments, given that the relative burden across these symptoms domains cannot be assumed in advance. Second, pain management protocols should integrate stress-specific interventions early in treatment, including mindfulness-based stress reduction, cognitive-behavioral stress management, and progressive muscle relaxation [46,47]. Third, multidisciplinary pain clinics should incorporate stress physiology education, teaching patients about the pain-stress cycle and providing concrete stress management tools. Addressing modifiable stress contributors identified in this study, including diabetes management and digital wellness interventions—may yield synergistic benefits.

From a public health perspective, these findings underscore the need for health literacy campaigns educating the public about sciatica prevention, early intervention importance, and the mind–body connection in pain. Community-based stress management programs, potentially integrated into primary care settings, could serve both preventive and therapeutic functions. Addressing social determinants of health through policies reducing income inequality and expanding educational opportunities may reduce both sciatica risk and associated psychological burden.

### 4.7. Strengths and Limitations

Study strengths include the large sample size exceeding minimum requirements, validated Arabic instruments, comprehensive assessment of sociodemographic and health factors, and rigorous multivariable modeling. However, several limitations warrant acknowledgment. The cross-sectional design precludes causal inference; longitudinal studies are needed to clarify temporal relationships. Sciatica diagnosis relied on self-reported physician diagnosis rather than clinical examination, potentially introducing misclassification. Important psychological mediators including pain, fear-avoidance beliefs, and coping strategies were not assessed. Additionally, the DASS-21 subscales are symptom severity scores derived from self-report and should not be interpreted as reflecting diagnostically distinct psychological mechanisms; the observed pattern of subscale differences requires confirmation using structured clinical interviews or formal diagnostic assessments before clinical or mechanistic conclusions can be drawn. The analysis did not apply formal correction for multiple testing across the three outcome models; given the exploratory nature of the study, findings should be interpreted with appropriate caution and require replication before clinical conclusions are drawn. Finally, we did not differentiate between acute and chronic sciatica presentations, pain severity levels, or treatment status. Additionally, several important potential confounders were not assessed, including pre-existing psychiatric history, comorbid chronic pain disorders, current pregnancy status, postpartum status, and neurological conditions. Furthermore, as both the exposure and all outcome measures were collected simultaneously via self-report, common-method bias may have artificially inflated observed associations. Finally, we did not differentiate between acute and chronic sciatica presentations, pain severity levels, or treatment status.

Furthermore, this study employed an online convenience sample recruited via social media, which may overrepresent younger, educated, and digitally active women; generalizability to older, rural, or less educated Saudi women should therefore be interpreted with caution. Future studies should employ stratified random sampling with offline recruitment components to obtain more representative samples, incorporate clinically confirmed sciatica diagnoses with standardized severity grading and clear temporal classification, and carefully distinguish sciatica from non-specific lower back pain in both measurement and analytic design. Additionally, sciatica and lower back pain share substantial symptom overlap and were included simultaneously in the multivariable models; their collinearity may limit the interpretive clarity of their independent associations with psychological outcomes.

## 5. Conclusions

This exploratory cross-sectional study found a pattern of higher stress symptom scores among Saudi women with self-reported sciatica compared to those without, while associations with depression and anxiety did not reach statistical significance. Additional factors associated with higher stress scores included lower back pain, diabetes, lower–middle income, and excessive phone use, while bachelor-level education was associated with lower stress scores. These findings should be interpreted cautiously given the self-report basis of both exposure and outcome measures, the convenience sampling strategy, and the absence of formal between-outcome comparisons. Future research employing clinically confirmed sciatica diagnoses, prospective designs, and representative sampling is needed to establish the nature and direction of these associations more rigorously.

## Figures and Tables

**Table 1:** Sociodemographic Characteristics of Study Participants (n = 706).

Variable	n	Mean (SD)/Proportion (%)
**Age (years)**	706	29 ± 11
**Height (cm)**	706	156 ± 7.1
**Weight (kg)**	706	59 ± 15
**BMI (kg/m^2^)**	706	24 ± 6.5
**Residence**	706	
Urban	305	43.2%
Village	401	56.8%
**Education**	706	
Secondary school or less	131	18.6%
Bachelor	542	76.8%
Postgraduate	33	4.7%
**Marital Status**	706	
Single	403	57.1%
Married	272	38.5%
Widowed/Divorced	31	4.4%
**Employment**	706	
Student	341	48.3%
Employed in governmental sector	165	23.4%
Unemployed	69	9.8%
Employed in private sector	24	3.4%
Retired	17	2.4%
Other	90	12.7%
**Family Monthly Income**	706	
Less than 5000 SAR *	250	35.4%
5000–9999 SAR	140	19.8%
10,000–14,999 SAR	144	20.4%
More than 15,000 SAR	172	24.4%

* SAR = Saudi Riyal (1 SAR ≈ 0.27 USD).

**Table 2:** Health-Related and Lifestyle Characteristics (n = 706).

Variable	n	Mean (SD)/Proportion (%)
**Smoking Status**	706	
Non-smoker	689	97.6%
Smoker	17	2.4%
**Physical Activity**	706	
Yes	415	58.8%
No	291	41.2%
**Comorbidities**		
Diabetes	706	
No	667	94.5%
Yes	39	5.5%
Arthritis	706	
No	586	83.0%
Yes	120	17.0%
**Daily Phone Use**	706	
Less than 2 h	39	5.5%
2–4 h	176	24.9%
4–8 h	289	40.9%
More than 8 h	202	28.6%
**Daily Car Driving**	706	
Less than 1 h	306	43.3%
1–2 h	263	37.3%
More than 2 h	137	19.4%
**Pregnancy History**	706	
Single/no pregnancy	439	62.2%
One time	35	5.0%
Two times	38	5.4%
Three times	43	6.1%
More than 3 times	151	21.4%

**Table 3:** Sciatica-Related Characteristics Among Participants (n = 706).

Variable	n	Proportion (%)
**Sciatica Diagnosis**	706	
No	628	88.9%
Yes	78	11.0%
**Sciatica Duration (among diagnosed, n = 78)**	78	
Less than one year	47	60.3%
More than one year	31	39.7%
**Lower Back Pain (LBP)**	706	
No	520	73.7%
Yes	186	26.3%
**Family History of Sciatica**	706	
No	492	69.7%
Yes	214	30.3%

**Table 4:** Distribution of Depression, Anxiety, and Stress Levels Among Participants (n = 706).

Variable	Category	n	Proportion (%)
**Stress**		706	
	Normal	541	76.6%
	Mild	57	8.1%
	Moderate	60	8.5%
	Severe	31	4.4%
	Extremely severe	17	2.4%
**Anxiety**		706	
	Normal	410	58.1%
	Mild	39	5.5%
	Moderate	134	19.0%
	Severe	36	5.1%
	Extremely severe	87	12.3%
**Depression**		706	
	Normal	463	65.6%
	Mild	62	8.8%
	Moderate	103	14.6%
	Severe	28	4.0%
	Extremely severe	50	7.1%

**Table 5:** Univariate Comparisons of Mental Health Scores by Key Variables.

Variable	Depression Score	*p*-Value	Anxiety Score	*p*-Value	Stress Score	*p*-Value
**Sciatica Diagnosis**						
No (n = 628)	11.2 ± 8.9	0.183	9.8 ± 8.7	0.481	13.8 ± 8.5	**<0.001**
Yes (n = 78)	12.8 ± 9.7		10.5 ± 9.3		21.4 ± 10.2	
**Lower Back Pain**						
No (n = 520)	11.0 ± 8.8	0.431	9.7 ± 8.6	0.423	13.2 ± 8.4	**0.001**
Yes (n = 186)	11.9 ± 9.4		10.3 ± 9.1		16.2 ± 9.8	
**Diabetes**						
No (n = 667)	11.2 ± 8.9	0.741	9.8 ± 8.7	0.519	13.9 ± 8.8	**0.004**
Yes (n = 39)	11.9 ± 10.1		10.8 ± 9.6		18.7 ± 11.2	
**Arthritis**						
No (n = 586)	11.0 ± 8.8	0.441	9.4 ± 8.5	**0.008**	13.6 ± 8.6	**0.003**
Yes (n = 120)	12.1 ± 9.6		11.8 ± 9.9		16.3 ± 10.1	
**Education Level**						
≤Secondary (n = 131)	12.1 ± 9.5	0.463	10.3 ± 9.2	0.522	15.8 ± 9.7	**0.015**
Bachelor (n = 542)	11.1 ± 8.8		9.7 ± 8.6		13.5 ± 8.6	
Postgraduate (n = 33)	11.5 ± 9.1		10.1 ± 9.0		14.2 ± 9.2	
**Family Income**						
<5000 SAR (n = 250)	11.8 ± 9.2	0.358	9.6 ± 8.5	0.588	14.9 ± 9.2	**0.031**
5000–9999 SAR (n = 140)	10.5 ± 8.3		9.5 ± 8.4		15.3 ± 9.1	
10,000–14,999 SAR (n = 144)	11.4 ± 9.0		10.2 ± 9.0		13.8 ± 8.7	
>15,000 SAR (n = 172)	11.2 ± 8.9		10.4 ± 9.1		12.9 ± 8.2	
**Daily Phone Use**						
<2 h (n = 39)	9.8 ± 7.8	0.555	8.9 ± 7.9	0.686	11.2 ± 7.3	**0.023**
2–4 h (n = 176)	10.9 ± 8.6		9.5 ± 8.3		13.1 ± 8.2	
4–8 h (n = 289)	11.3 ± 9.1		9.8 ± 8.8		14.2 ± 8.9	
>8 h (n = 202)	11.8 ± 9.2		10.3 ± 9.1		15.1 ± 9.5	

Values presented as mean ± standard deviation. Bold *p*-values indicate statistical significance (*p* < 0.05). Independent samples *t*-tests used for binary variables; ANOVA for categorical variables with >2 levels. SAR = Saudi Riyal.

**Table 6:** Multivariable Linear Regression Predictors of Stress (n = 706).

Predictor	β	95% CI	*p*-Value
**Sciatica-Related Factors**			
Sciatica diagnosis (vs. no)	6.87	4.57 to 9.17	**<0.001**
Lower back pain (vs. no)	2.15	0.60 to 3.70	**0.007**
**Chronic Conditions**			
Diabetes (vs. no)	3.13	0.21 to 6.05	**0.036**
Arthritis (vs. no)	0.74	−1.33 to 2.82	0.483
**Socioeconomic Factors**			
Education			
Bachelor (vs. ≤secondary)	−2.21	−3.99 to −0.43	**0.015**
Postgraduate (vs. ≤secondary)	−1.28	−4.94 to 2.37	0.491
Family Monthly Income			
5000–9999 SAR (vs. <5000)	2.11	0.20 to 4.03	**0.031**
10,000–14,999 SAR (vs. <5000)	0.91	−1.06 to 2.88	0.363
>15,000 SAR (vs. <5000)	0.29	−1.57 to 2.15	0.758
**Lifestyle Factors**			
Daily Phone Use			
2–4 h (vs. <2 h)	0.17	−1.53 to 1.88	0.841
4–8 h (vs. <2 h)	0.17	−1.53 to 1.88	0.841
>8 h (vs. <2 h)	2.17	0.29 to 4.04	**0.023**
Physical activity (vs. no)	−0.15	−1.51 to 1.21	0.826
Smoking (vs. no)	−0.12	−4.49 to 4.25	0.958
**Demographic Factors**			
Age (per year)	−0.05	−0.17 to 0.07	0.431
BMI (per kg/m^2^)	−0.03	−0.15 to 0.09	0.626
Rural residence (vs. urban)	−0.55	−1.89 to 0.79	0.420
Marital Status			
Married (vs. single)	1.06	−2.26 to 4.39	0.530
Widowed/divorced (vs. single)	3.58	−0.74 to 7.89	0.104
Employment			
Governmental sector (vs. unemployed)	0.08	−2.20 to 2.37	0.942
Private sector (vs. unemployed)	2.60	−1.26 to 6.46	0.186
Student (vs. unemployed)	1.88	−0.38 to 4.14	0.103

Note: Bold text indicates statistical significance (*p* < 0.05). Model adjusted for all variables listed.

## Data Availability

The data presented in this study is available upon request from the corresponding author. The data is not publicly available due to ethical restrictions and privacy concerns related to sensitive health information.

## Abbreviations

The following abbreviations are used in this manuscript:

BMI	Body Mass Index
LBP	Lower Back Pain
SAR	Saudi Riyal
DASS-21	Depression, Anxiety, and Stress Scale (21-item)

## Appendix A

**Table A1:** Multivariable Linear Regression Analysis of Predictors of Depression (n = 706).

Predictor	β	95% CI	*p*-Value
**Sciatica-Related Factors**			
Sciatica diagnosis (vs. no)	1.80	−0.85 to 4.44	0.183
Lower back pain (vs. no)	0.72	−1.07 to 2.50	0.431
**Chronic Conditions**			
Diabetes (vs. no)	0.57	−2.79 to 3.92	0.741
Arthritis (vs. no)	0.94	−1.45 to 3.33	0.441
**Socioeconomic Factors**			
Education			
Bachelor (vs. ≤secondary)	0.77	−1.28 to 2.82	0.463
Postgraduate (vs. ≤secondary)	0.95	−3.26 to 5.16	0.658
Family Monthly Income			
5000–9999 SAR (vs. <5000)	−1.03	−3.24 to 1.17	0.358
10,000–14,999 SAR (vs. <5000)	0.78	−1.48 to 3.05	0.497
>15,000 SAR (vs. <5000)	−0.26	−2.40 to 1.88	0.810
**Lifestyle Factors**			
Daily Phone Use			
2–4 h (vs. <2 h)	0.11	−1.85 to 2.07	0.909
4–8 h (vs. <2 h)	0.11	−1.85 to 2.07	0.909
>8 h (vs. <2 h)	0.65	−1.51 to 2.80	0.555
Physical activity (vs. no)	−0.58	−2.15 to 0.98	0.464
Smoking (vs. no)	3.11	−1.91 to 8.14	0.224
**Demographic Factors**			
Age (per year)	0.05	−0.09 to 0.19	0.485
BMI (per kg/m^2^)	0.07	−0.06 to 0.21	0.276
Rural residence (vs. urban)	0.07	−1.48 to 1.61	0.930
Marital Status			
Married (vs. single)	−0.98	−4.81 to 2.85	0.615
Widowed/divorced (vs. single)	3.75	−1.21 to 8.72	0.138
Employment			
Governmental sector (vs. unemployed)	−2.43	−5.06 to 0.20	0.070
Private sector (vs. unemployed)	−2.42	−6.86 to 2.02	0.285
Student (vs. unemployed)	−0.86	−3.46 to 1.74	0.514
Driving per Day			
1–2 h (vs. <1 h)	−0.64	−2.42 to 1.13	0.477
>2 h (vs. <1 h)	−0.98	−3.13 to 1.16	0.369
Number of Pregnancies			
One time (vs. none/single)	0.86	−3.86 to 5.59	0.719
Two times (vs. none/single)	3.72	−0.94 to 8.39	0.117
Three times (vs. none/single)	−0.32	−4.86 to 4.21	0.889
>3 times (vs. none/single)	−1.64	−5.53 to 2.24	0.407

**Table A2:** Multivariable Linear Regression Analysis of Predictors of Anxiety (n = 706).

Predictor	β	95% CI	*p*-Value
**Sciatica-Related Factors**			
Sciatica diagnosis (vs. no)	0.45	−0.80 to 1.70	0.481
Lower back pain (vs. no)	0.34	−0.50 to 1.18	0.423
**Chronic Conditions**			
Diabetes (vs. no)	0.52	−1.06 to 2.10	0.519
Arthritis (vs. no)	**1.52**	**0.39 to 2.64**	**0.008**
**Socioeconomic Factors**			
Education			
Bachelor (vs. ≤secondary)	0.32	−0.65 to 1.28	0.522
Postgraduate (vs. ≤secondary)	0.87	−1.12 to 2.85	0.391
Family Monthly Income			
5000–9999 SAR (vs. <5000)	−0.29	−1.33 to 0.75	0.588
10,000–14,999 SAR (vs. <5000)	0.39	−0.67 to 1.46	0.470
>15,000 SAR (vs. <5000)	0.58	−0.43 to 1.59	0.260
**Lifestyle Factors**			
Daily Phone Use			
2–4 h (vs. <2 h)	0.03	−0.90 to 0.95	0.951
4–8 h (vs. <2 h)	0.03	−0.90 to 0.95	0.951
>8 h (vs. <2 h)	0.21	−0.81 to 1.22	0.686
Physical activity (vs. no)	0.02	−0.71 to 0.76	0.948
Smoking (vs. no)	1.32	−1.05 to 3.69	0.276
**Demographic Factors**			
Age (per year)	0.03	−0.04 to 0.09	0.435
BMI (per kg/m^2^)	0.02	−0.05 to 0.08	0.618
Rural residence (vs. urban)	0.12	−0.61 to 0.84	0.754
Marital Status			
Married (vs. single)	−0.17	−1.98 to 1.63	0.851
Widowed/divorced (vs. single)	1.51	−0.83 to 3.85	0.206
Employment			
Governmental sector (vs. unemployed)	−0.58	−1.82 to 0.66	0.355
Private sector (vs. unemployed)	−0.93	−3.02 to 1.16	0.384
Student (vs. unemployed)	0.37	−0.85 to 1.60	0.550
Driving per Day			
1–2 h (vs. <1 h)	−0.18	−1.02 to 0.66	0.674
>2 h (vs. <1 h)	−0.43	−1.44 to 0.58	0.404
Number of Pregnancies			
One time (vs. none/single)	0.44	−1.79 to 2.66	0.699
Two times (vs. none/single)	1.73	−0.47 to 3.93	0.122
Three times (vs. none/single)	0.13	−2.01 to 2.27	0.903
>3 times (vs. none/single)	−1.47	−3.30 to 0.36	0.116

Note: Bold text indicates statistical significance (*p* < 0.05). SAR = Saudi Riyal. Model adjusted for all variables listed.
